# YummyData: providing high-quality open life science data

**DOI:** 10.1093/database/bay022

**Published:** 2018-03-09

**Authors:** Yasunori Yamamoto, Atsuko Yamaguchi, Andrea Splendiani

**Affiliations:** 1 Database Center for Life Science, Research Organization of Information and Systems, Kashiwa, Japan; 2 Novartis Institutes for Biomedical Research, Basel, Switzerland

## Abstract

Many life science datasets are now available via Linked Data technologies, meaning that they are represented in a common format (the Resource Description Framework), and are accessible via standard APIs (SPARQL endpoints). While this is an important step toward developing an interoperable bioinformatics data landscape, it also creates a new set of obstacles, as it is often difficult for researchers to find the datasets they need. Different providers frequently offer the same datasets, with different levels of support: as well as having more or less up-to-date data, some providers add metadata to describe the content, structures, and ontologies of the stored datasets while others do not. We currently lack a place where researchers can go to easily assess datasets from different providers in terms of metrics such as service stability or metadata richness. We also lack a space for collecting feedback and improving data providers’ awareness of user needs. To address this issue, we have developed YummyData, which consists of two components. One periodically polls a curated list of SPARQL endpoints, monitoring the states of their Linked Data implementations and content. The other presents the information measured for the endpoints and provides a forum for discussion and feedback. YummyData is designed to improve the findability and reusability of life science datasets provided as Linked Data and to foster its adoption. It is freely accessible at http://yummydata.org/.

Database URL: http://yummydata.org/

## Introduction

Modern life science research is very data-intensive: to understand the functions of biological systems, scientists rely on a variety of data about the systems and their functions. These data are very heterogeneous in scope, ranging from molecular mechanisms to phenotypes and beyond. The ways it is generated and collected are also varied, as they have been assembled over time by large and small scientific investigations, deposited in large institutional repositories and conveyed in publications.

The current life science literature includes about 27 million papers in PubMed, genomic databases storing 200 million sequences in GenBank (https://www.ncbi.nlm.nih.gov/genbank/statistics/), and pathway data scattered over at least 165 databases (http://www.oxfordjournals.org/nar/database/cap/). This is a very large and complex information landscape whose exploitation is one of the keys to a data-driven approach to science.

Exploiting such scattered information requires taking an integrated view of the data, which in turn requires locating relevant data and making it accessible in a way that facilitates integration. This is a complex task, not only because of the intrinsic nature of the information itself, but also because the same information can be delivered by a variety of providers, with different data formats, terminologies, and update policies. In addition, many datasets aggregate other data sources, in more or less indirect ways, so the provenance of the dataset itself can be hard to delineate.

In order to cope with such a wide range of representations and formats, approaches based on Linked Data have been proposed (1) and now partially adopted (2). These approaches (presented in more detail later) provide means of using ontologies to describe data, as well as a standard language (the Resource Description Framework, or RDF) and access protocol (SPARQL). In addition to Linked Data, the Findable, Accessible, Interoperable and Reusable (FAIR) initiative (3) has proposed a broader set of requirements to enable life science data interoperability.

The RDF and Linked Data formats have significant roles to play in allowing heterogeneous databases, scattered around the world, to be used in an integrated manner. In RDF, entities are identified via global identifiers that can be resolved over the Internet using Web technologies. Once resolved, relevant information is provided in standard formats, making use of standard predicates that also provide explicit links to other entities and datasets. This combination enables information to easily be retrieved and merged according to a unified (albeit schema-light) model. An example of this approach that combines information about a gene, the protein it produces, related diseases, and references to relevant papers can be found in (2).

The RDF and Linked Data formats are being adopted by variety of large and small dataset providers. To cite a few relevant examples, the European Bioinformatics Institute (EBI) provides their major databases including UniProt in RDF (4), in addition to legacy data representations such as text files. Similarly, the National Center for Biotechnology Information provides RDF versions of MeSH (5) and PubChem (6). The Database Center for Life Science (DBCLS) has constructed several RDF datasets in cooperation with the National Bioscience Database Center (NBDC) and has set up a portal site called the NBDC RDF Portal (https://integbio.jp/rdf/) to disseminate them.

That said, the impact of such Linked Data approaches is being severely limited by datasets being published and republished without any real quality control. To illustrate this, imagine that we are interested in finding a representation of an apoptosis pathway. This information is available in different representations from different providers, such as GO (7), Reactome (8) and BioCyc (9), which in turn often derive their datasets from the literature, through more or less curated processes.

The datasets delivered by these providers are then further integrated and republished by systems such as UniProt (10) and PathwayCommons (11), which may introduce additional data or normalization steps. A single dataset can be published by multiple providers, in various (not always explicitly distinguished) versions. For instance, PathwayCommons, the EBI RDF platform (4), OpenPHACTS (12) and Linked Life Data (http://linkedlifedata.com/) provide different versions of data from the Reactome database. Most of these providers deliver datasets in RDF and even release SPARQL access points, which would be easy to process if we were interested in retrieving all genes associated with apoptosis. However, if the sites provide different data, which one should we use? Short of just merging all the data, there is no easy way to determine which dataset to use.

In this article, we propose a system that monitors SPARQL endpoints and the datasets accessible through them to help assess and improve the quality of the data provided via Linked Data technologies. (Hereafter, the term ‘endpoint’ will be used to mean ‘SPARQL endpoint’ unless otherwise noted.) We believe that the current lack of such assessment is preventing us from realizing the full benefits of providing such data via RDF and hence limiting our ability to undertake data-driven research efficiently. To remedy this, we introduce the Umaka Score, a simple index for quality assessment. (‘Umaka’ is a Japanese dialect word that means ‘yummy’ in English.)

In addition, we propose a discussion space (i.e. a forum) where data providers and consumers can communicate with each other to facilitate mutual understanding and improve data usability in the life science community. As YummyData collects ‘evidence’ about a number of endpoint features, it is a natural place to start evidence-based discussions about missing features or to clarify other points.

In this article, we first introduce the design of YummyData, its features, and how they will hopefully help to address the RDF adoption issues that we have identified. We then provide a brief review of related work. As monitoring can be seen as an extension of indexing, we provide a few examples illustrating the evaluation of systems for indexing and monitoring scientific data, including YummyData. Next, we provide details of both the technical implementation of our system and the metrics and scores computed by it. We then present the results that YummyData has already delivered. Finally, we discuss YummyData’s role in supporting innovation, relating it to other work and using a simple story to clarify the issues currently faced by researchers attempting to leverage the potential of Linked Data for life science research.

## YummyData design

The goal of YummyData is to improve the usability of Linked Data for life science research. From the consumer’s perspective, it is important to be able to find the right data source, as well as easily accessible information about how it can be used (e.g. license or ontology information). From the provider’s perspective, it is important to receive recognition for their investment in maintaining a valuable dataset and keeping it up-to-date. It is also important to gather consumer feedback.

As a result, we have designed YummyData as a multi-faceted resource. In particular, YummyData can be seen as a monitoring server, an information point for both consumers and providers, a scoring system, a discussion space, and a service for software agents.

### YummyData as a monitoring service

YummyData implements a monitoring service that periodically evaluates several parameters of each endpoint. Some of these parameters are related to the endpoint’s content (e.g. the number of triples stored) and some to the operation of the triplestore (e.g. uptime). YummyData maintains a historic log of this information.

There are two reasons for this data monitoring. On the one hand, it collects information about the endpoints that can be served to users and consumers (see below). On the other hand, having a historic log of responses also provides evidence of an endpoint’s reliability and the changes in its content. This information is the foundation on which other the aspects of YummyData are built.

### YummyData as an information point

YummyData acts as an information point for both consumers and providers. For consumers, it provides both aggregate views of the state of the Linked Data information and specific information for each endpoint.

**Figure 1.**
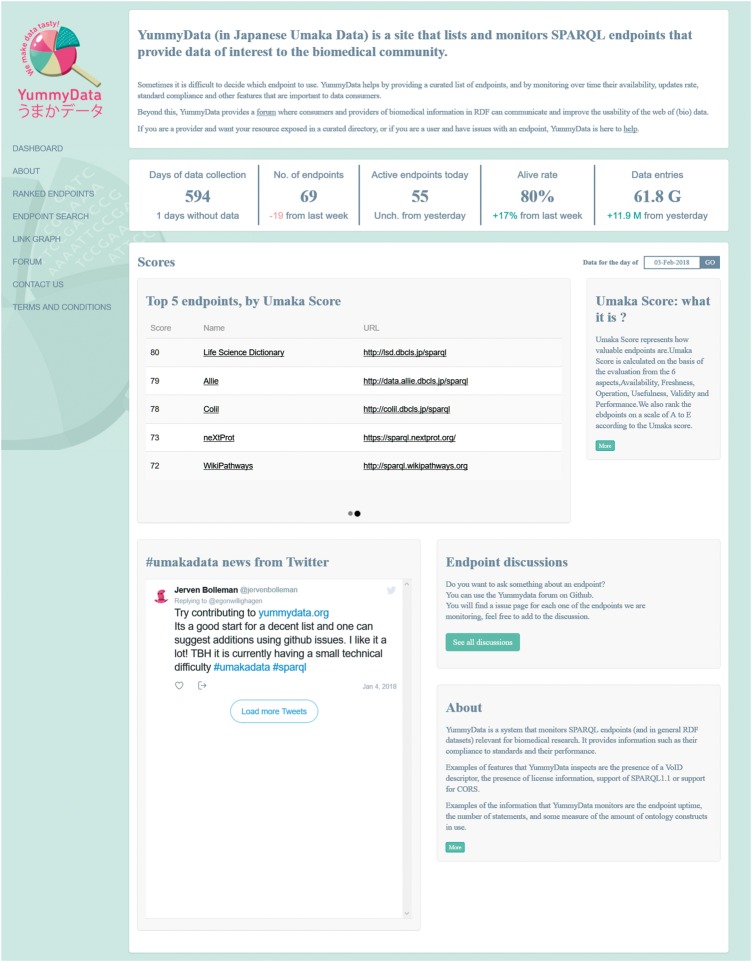
The dashboard page.

For the aggregate views, YummyData first provides a list of the endpoints being monitored (65 at the time of writing). Originally, this list was derived automatically from the Comprehensive Knowledge Archive Network (CKAN), but over time we have come to opt for a more curated approach that better serves the needs of life science researchers (e.g. removing less useful and poorly maintained endpoints while adding valuable endpoints to the index). The global state of the monitored endpoints is then presented via a dashboard page (Figure 1), which gives summary information such as the total number of endpoints, the number of days when data has been available, alive rate, total number of triples monitored, and a summary the current scores (detailed below). The list of endpoints itself is presented as a ranked list (according to the scores explained below).

**Figure 2.**
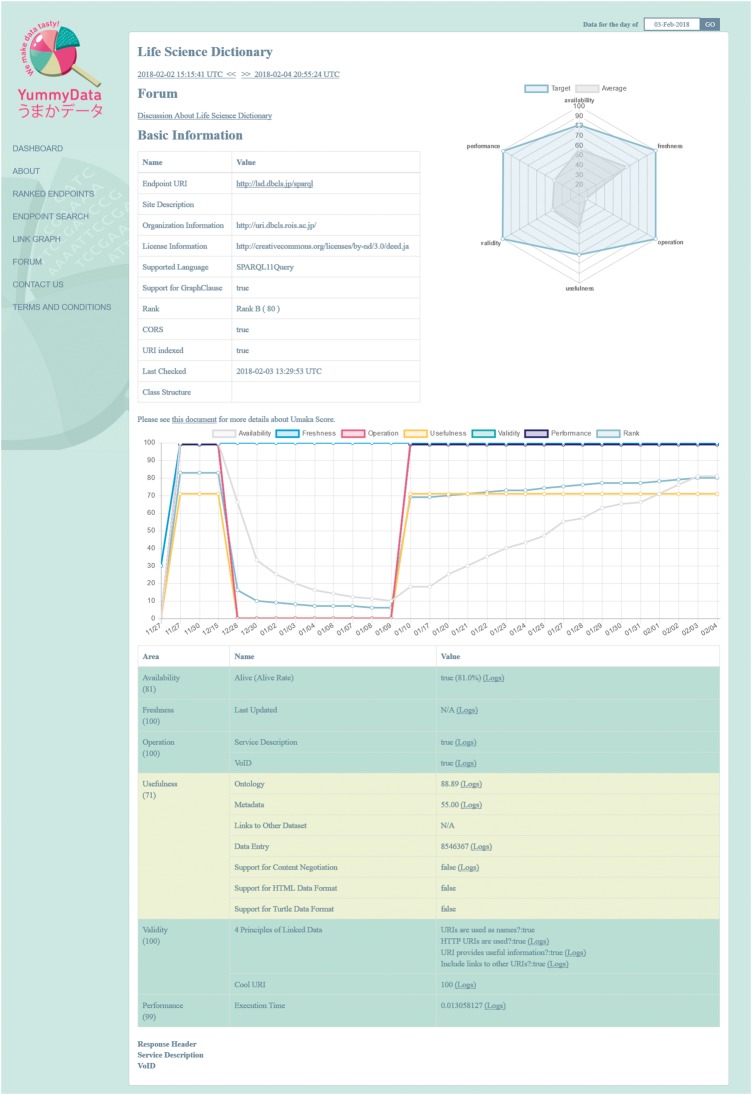
An endpoint information page.

For each endpoint, we present information covering six aspects of data quality, as defined at http://yummydata.org/umaka-score.html: Availability, Freshness, Operation, Usefulness, Validity and Performance (see the “Material and methods” section for further details). This is complemented by additional useful information: the license under which the dataset is provided, whether it supports SPARQL 1.1 (and Cross-Origin Resource Sharing, CORS), and both the service and VoID descriptors (Figure 2). A search function allows the endpoints to be filtered according to several criteria (which are mirrored in the API for programmatic access). All the information provided by YummyData can be accessed for any day when the endpoint was monitored.

### YummyData as an information point for providers

Logs are kept for all the above measurements, together with the request/response headers that generated the values. This information is useful for debugging endpoint problems detected by YummyData. YummyData also provides a summary of how the endpoint in question compares to the average endpoint, which providers can use to help understand how their implementation compares with those of their peers.

### YummyData as a scoring system

Maintaining a service such as an endpoint is an expensive exercise, and most current providers are academic or institutional providers that maintain open, public datasets. There is no direct reward for their investment in providing a high-quality service, nor any way to make this investment visible.

**Figure 3.**
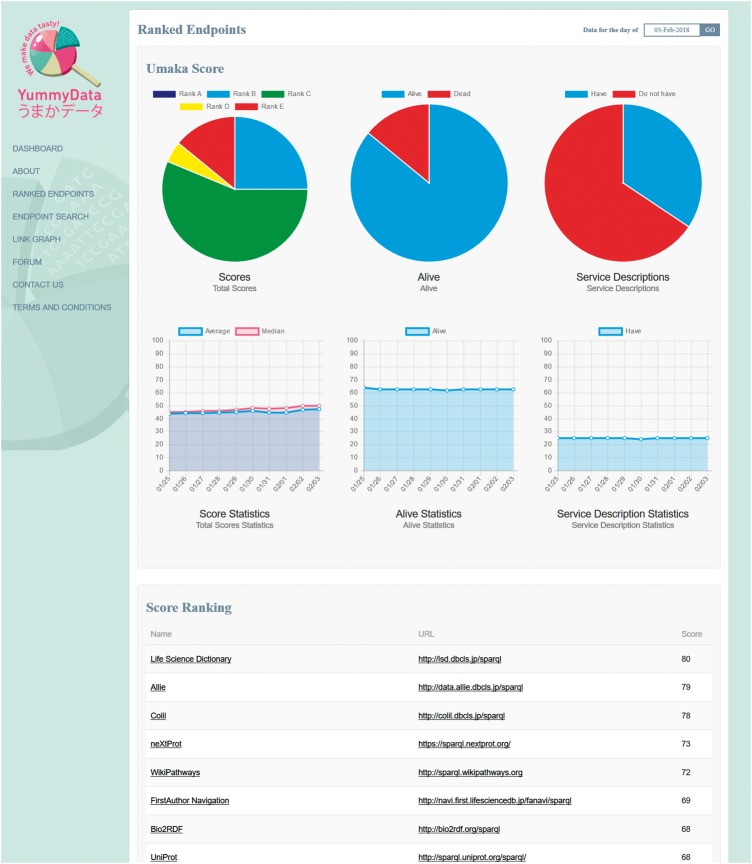
The ranked list page.

In order to provide an incentive to providers, YummyData implements a simple scoring system: all the measurements taken are combined into a single ‘Umaka Score’ that is used to rank the endpoint on our lists (Figure 3). There is a degree of arbitrariness in how this score is defined; both in deciding what should contribute to the score and in deriving a single metric from a variety of discrete and continuous measurements, some of which are only approximate. That said, we believe that providing a simple score helps draw attention to the quality of the data provision.

We expect that, when their score indicates poor performance, providers may investigate the ranking method and find some flaw in the scoring algorithm. As all our measurements (queries and responses) are publicly available, we believe this represents valuable and virtuous feedback, as it incentivizes providers to find accidental errors in the implementation and contribute to refining the score itself. The score is defined as the average of the scores for six individual aspects, each of which ranges from 0 to 100. Detailed specifications of the individual aspect scores are available at http://yummydata.org/umaka-score.html.

### YummyData as a discussion space

Another important feature of YummyData is that it provides a discussion space for each endpoint. Since YummyData collects ‘evidence’ about a variety of endpoint features, this offers a natural place to discuss missing features or clarify other points. At the time of writing, open issues include requests for new endpoints to be listed, requests for particular scores to be added, and discussions on issues found with specific endpoints.

Admittedly, this aspect of YummyData is still relatively new and underused, but this depends on the endpoint’s prominence, and the feedback gathered so far has been limited to times when an early endpoint prototype was presented. As we publicize this feature more widely, we expect that it will increasingly be used as a community resource.

### YummyData as a service for agents

YummyData also makes its information available to software agents via an API.

YummyData’s API allows endpoints to be selected based on particular criteria and data to be retrieved about them. The endpoint selection criteria include Umaka Score or rank, features like uptime and more. In addition, the information that can be retrieved includes not just the score but more detailed data, both present and past, complete with timestamps.

YummyData can thus provide quasi-real-time information on endpoint status and content to other systems, for instance to agents that need to decide how to execute a Linked Data query.

## Related works

The need to index scientific knowledge is not new: PubMed itself, as well as several other public repositories, were designed to address this need. However, the increasing number of datasets provided has created an additional need for a catalog of available resources.

One example of an index rooted in the traditional scientific publication process is the NAR database issue. In 1998, the NAR journal started to provide a listing of available resources as a special yearly database issue. Now, it also maintains a list of databases enriched with metadata: the NAR online Molecular Biology Database Collection (http://www.oxfordjournals.org/nar/database/a/), which currently indexes 1615 databases. The 2017 NAR database issue includes 152 database papers, 54 introducing new databases and 98 describing updates to existing databases (13).

With so many resources to choose from, an automated approach to analyzing and integrating data becomes necessary. The NAR database issue is limited in this regard, as it describes databases in natural language and the information in the papers is not provided in a machine-readable form.

Although the metadata for the databases in the NAR online Molecular Biology Database Collection is provided as structured data, it only includes the URL, a contact point, a short description, and references for each database.

Whereas NAR relies on curators to identify and describe datasets, other sources instead rely on crowdsourcing and are generally not limited to a specific domain. One such example is DataHub (https://datahub.io/), a CKAN-based platform for indexing datasets (14), which 

offer advanced features such as version management and providing metadata in various formats. As of January 2017, it includes 11 110 datasets annotated with structured metadata.

Although DataHub’s coverage is rich, its precision is limited: because the datasets and associated metadata are updated manually by their ‘owners’, the information can easily become out-of-date (e.g. when a system is shut down, DataHub is often not notified).

Other approaches to indexing life science information have taken Web-centric approaches. Sindice (15) was a service for searching semantic metadata embedded in Web pages that had an index of over 700 M pages, generated by crawling 20 M pages per day. Sindice allowed applications to retrieve sources of a given resource automatically, together with information about them. Sindice ceased operating in 2014.

A more recent approach to indexing based on search engines is schema.org, a set of standard terminologies for annotating pages (e.g. via microformats) that was initially developed jointly by major industry players (e.g. Google, Bing and Yahoo). Search engines can utilize the annotations standardized by schema.org to improve search, both to improve discoverability and structure the results. BioSchemas (http://bioschemas.org/) builds on schema.org by providing a set of specialized metadata for life science information.

In the narrower field of information provided as Linked Data, LODStats (http://stats.lod2.eu/) periodically crawl sites to download datasets or endpoints to obtain statistics such as the numbers of triples, entities, and literals. As of May 2017, it encompasses 9960 datasets registered on DataHub, PublicData.eu and data.gov.

For information provided as Linked Data, SPARQLES (http://sparqles.ai.wu.ac.at/) is a richer and more focused resource that monitors SPARQL endpoints registered in DataHub to determine their availability, performance, interoperability, and discoverability.

YummyData complements these initiatives by providing a resource with a broader set of goals (support for both providers and consumers) as well as an operational focus on life science resources.

Unlike NAR, we focus on datasets that are amenable to computational integration (i.e. which supply RDF metadata) and provide processable support metadata (e.g. which version of what ontology is being used). Unlike DataHub, we provide a curated list of data sources, based on both human input (e.g. selection of relevant endpoints) and continuous monitoring. Unlike Sindice and schema.org, we focus not on search but rather on assessing the overall quality of the data provision. We also have a different focus than LODStats: our goal is the statistical analysis of datasets. We focus on compliance with Linked Data principles and thus collect additional information (e.g. whether there is support for content negotiation or dereferenceable URIs).

SPARQLES is the service that is closest to YummyData in spirit. However, with YummyData we took a few additional steps that, we believe, will make it a more valuable resource for computational life science researchers and the overall life science Linked Data community.

In particular, we focus on a curated list of endpoints, rather than an automatically discovered one, as our goal is to provide useful information to researchers, not comprehensively index all available datasets.

We are also not limited to providing information to consumers: we provide information (e.g. detailed output logs from the endpoints) that is intended to help providers improve their services. We provide a different set of metrics for consumers that attempt to measure aspects of data quality (e.g. refresh rate) and usability (e.g. use of shared ontologies) that go beyond the endpoint implementation.

Finally, we also provide community tools: a scoring system to incentivize provider investment and a forum where we hope data providers and consumers can discuss the endpoint scores with a view to developing more useful resources.

## Materials and methods

### YummyData implementation

As discussed earler, YummyData consists of two parts: a crawler that collects the data used to calculate the Umaka Scores and a Web service that makes the calculated scores and other data available via a Web page and through an API. Both components were developed using Ruby (Ruby on Rails for the Web service) and are released as Docker images (https://www.docker.com) that can be easily deployed on different hosts. The complete source code can be freely accessed from our GitHub repository (https://github.com/dbcls/umakadata).

#### The YummyData crawler

The crawler runs as a daily cron job, and the data obtained are stored in a PostgreSQL database. The crawler issues a series of HTTP queries, some based on SPARQL and some not. The non-SPARQL queries include HTTP GET requests to locate VoID (https://www.w3.org/TR/void/) and SPARQL 1.1 Service Descriptions (SDs) (https://www.w3.org/TR/sparql11-service-description/), as well as general metadata provided by the endpoint. All queries and responses (including response codes) are retained, in order to provide the best possible evidence for the information provided.

If a query fails, it is not retried, to minimize the burden on the target endpoints as much as possible. As data are collected daily, we can compensate for any failures by looking at the history of each endpoint. More generally, we follow special procedures to mitigate the burden of issuing SPARQL queries to endpoints containing large numbers of triples (i.e. more than one billion). Our crawler can easily be identified by its HTTP User-Agent header: ‘Umaka-Crawler/1.0 by DBCLS (umakadata@dbcls.jp)’.

#### YummyData website and API

YummyData’s Web component provides both Web views and an API for the collected (and computed) information; the public-facing Web views were described earlier (both the dashboard and endpoint-specific pages). YummyData also provides an access-controlled administration page where administrators can add or remove endpoints from the list, and edit their names, endpoint URLs, and related websites. In addition, administrators can check when a given endpoint was most recently crawled. If the crawler fails to obtain data from an endpoint for >30 consecutive days, it raises a dead flag to suggest that the administrators should consider removing the endpoint.

Detailed API specifications are available online (http://yummydata.org/api/specifications). The API returns data in the JSON and JSON-LD formats.

It should be noted that, as obtaining data from all the endpoints takes more than a day, the most recent results provided on the YummyData pages are based on data obtained 2 days previously.

The YummyData scores are computed on demand at query time (both for Web- and API-based requests). This allows us to modify our endpoint scoring algorithm and have the results be reflected immediately by all YummyData services. Since our scoring method is rather subjective, the system needs to be flexible enough to allow the scoring approach to adapt and evolve, especially since the service is still in its early stages.

### Endpoint list determination

To find endpoints relevant to the life sciences, we use DataHub (https://datahub.io/), related publications, Google searches and direct input (e.g. via forums). We then remove inactive endpoints: if the crawler fails to obtain data for >30 consecutive days, we check whether the service has been discontinued and remove it from our list.

At the time of writing, YummyData is tracking 65 endpoints (as well as 19 endpoints for which the crawler has failed to obtain data for >30 consecutive days, but which have not been confirmed as permanently discontinued).

### Computing the quality metrics and Umaka Scores

Our system computes endpoint metadata that highlight six aspects of data quality: Availability, Freshness, Operation, Usefulness, Validity, and Performance. These are then combined to calculate the Umaka Scores that are used to rank the endpoints in our lists.

The availability or alive rate, of an endpoint is calculated by dividing the number of days, out of the last 30, when the crawler succeeded in accessing the server by 30. Success here means that the server responded with an HTTP status code of 200.

In order to provide an informed estimate of the Freshness of the dataset obtainable through an endpoint, we track the number of triples over time, using changes in this number as a proxy for updates, which in turn are a proxy for the dataset’s freshness. Although the number of triples staying constant over time does not necessarily imply that the system is not being updated, this data, combined with versioning and update information from the VoID descriptor, allows users to reasonably estimate the dataset’s freshness. As only a few data providers include update information in their VoID descriptors, we usually obtain this data by issuing SPARQL queries, except for some special cases where this is not feasible.

The Operation of the service (beyond its uptime) is captured by the provision (or lack thereof) of standard metadata, such as the SD and VoID.

A variety of information is captured to determine the endpoint’s Usefulness, ranging from the availability of information in different formats (e.g. Turtle and HTML) via content negotiation to metrics reflecting the modeling and interlinkage of the provided data. In particular, an ‘ontology’ metric reflects the number of ontologies declared in the datasets and the usage of ‘standard’ vocabularies. We consider vocabularies to be ‘standard’ if they are defined in the LOV (http://lov.okfn.org/) or in commonly-used ontologies within the life science domain. (Not all life science ontologies are found in the LOV; e.g. FALDO (16) was missing when YummyData was first released. It has then been added as our system helped reveal its omission). A ‘metadata’ metric instead reflects the extent to which entities are annotated with classes, labels, or datatypes.

The Validity of an endpoint reflects its adoption of the Linked Data guiding principles (https://www.w3.org/DesignIssues/LinkedData.html), especially whether URIs are resolvable and provide links to other URIs. We also monitor whether they follow ‘cool URI’ guidelines (https://www.w3.org/TR/cooluris/). We manually check the prefixes to determine whether or not each URI is owned by the provider, since it is nontrivial to annotate each URI with its owner.

Finally, Performance information is captured, essentially based on query execution time (compensating for latency).

The above metrics are complemented by additional useful information: the license under which the dataset is provided, whether the endpoint supports SPARQL 1.1 (and CORS), and both the service and VoID descriptors.

### Forum

We use GitHub’s Issues to provide a forum. Each endpoint has its own label and all GitHub users are free to post their comments. When a YummyData administrator adds a new endpoint to be monitored, a corresponding label is created and a new Issue posted with that label. All Issues related to YummyData can be accessed at https://github.com/dbcls/LinkedData-Agora/issues.

## Results

YummyData was first launched in 2012 as a result of BioHackathon 2012 (17), but it was initially unstable due to insufficient maintenance and development time. After reaffirming its importance, the service was later revived and extended, and was relaunched in March 2016 covering 18 endpoints. As of 27 June 2017, it monitors 65 endpoints, holds 452 days of detailed historic data, and has been accessed 2484 times since December 2016.

The system demonstrates how the same datasets can be offered by multiple providers but with different levels of support. For instance, Reactome is provided by the EBI RDF platform, Bio2RDF and Pathway Commons, while ChEBML is provided by the EBI RDF platform, Bio2RDF, Uppsala University and DrugBank. The information in YummyData helps users to assess which of these endpoints are better suited to their needs.

A few applications have already been built that rely on YummyData services, such as SPARQL Builder (18). This system conducts SPARQL queries in response to user searches for datasets of interest, and can check the alive rates and response times of SPARQL endpoints to avoid dead or unreliable endpoints before constructing user queries.

LODInspector is a simple prototype, developed at BioHackathon 2016 (https://github.com/sgtp/LodInspector) that looks for triples about entities that can be found in the LOD, conducting some rudimentary query expansion and overlap analysis. It uses YummyData to dynamically determine which endpoints to query.

It should be noted that YummyData has so far only been publicized as a beta in restricted circles, so its impact, in terms of community feedback, is still limited.

Despite this, we already have anecdotal evidence that the NBDC in Japan improved their NBDC RDF Portal endpoint to provide SD based on feedback from YummyData. The community forums have also already proved to be valuable: at the time of writing, open issues include requests for new endpoints to be listed, requests for particular scores to be added, and discussion of issues found with specific endpoints.

During August 2017, we obtained the following results for 65 endpoints.

- Average alive rate: 96.3%
- Average Umaka Rank: 3.13 (1:E–5:A)
- Average rate of SD provision: 24.3%
- Average rate of VoID provision: 8.87%

The reason why the alive rate is so high is that we have eliminated the endpoints that had been inaccessible for >30 consecutive days. This shows that few endpoints frequently go down and then come up again over the course of a month.

## Discussion

### YummyData, innovation and FAIR

The idea that all published data should be accessible and well annotated to support on-demand data mining is perhaps the holy grail of bioinformatics. Several initiatives have pushed for the publication of scientific data in a machine-readable and open way or, in other words, to make data FAIR (3).

The adoption of Linked Data principles has the potential to make data FAIR and change the way life science research is performed, but such innovation requires both invention and adoption. Although the Linked Data principles are a valuable invention, their adoption is still patchy, despite the fact that the role of Linked Data in supporting life science research was proposed as long ago as 2000, that some relevant examples of integrated knowledgebases were presented in 2007 (19), and that we now have a significant amount of data available.

### An example illustrating the uptake of linked data, and its limits

There are many possible explanations for the slow uptake of these technologies. One is that, as they rely on precise semantic characterization of information, they are limited by our ability to describe data with coherent, shared terminologies, or ontologies. To a degree, the adoption of Linked Data in the life sciences is accompanying the development of a unified (computational) language for this broad area of science, and this process takes time. Another explanation is that while large-scale data-driven research may be the future, most current research is still reductionist in nature, so the data integration capabilities offered by Linked Data technologies are not yet needed by the bulk of researchers.

That said, there is also a third explanation for this slow adoption, one that we intend to tackle with YummyData: RDF and Linked Data are supposed to assist the data integration process. If this process is made harder by the difficulty of finding ‘good’ RDF datasets, then the costs of using these technologies may outweigh their benefits.

**Figure 4.**
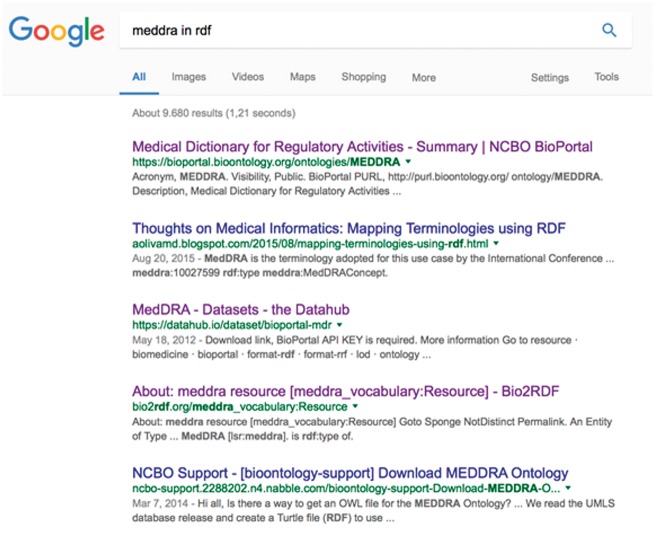
The ranked list obtained by searching Google for ‘MedDRA in RDF’.

To illustrate this point, we now consider a simple real-life example of the issues that researchers face in searching for RDF datasets. As a typical use case, imagine that a bioinformatician is looking for a version of MedDRA (https://www.meddra.org/) in RDF. Adverse events (relative to the overall data associated with a drug) are often annotated via a standardized terminology called MedDRA. MedDRA itself is structured as a hierarchy, so combining the MedDRA classification with the actual data would enable queries such as, ‘find all adverse events for a given compound in a given systemic area’. The first thing the researcher would be likely to do would be to just search the Web for ‘MedDRA in RDF’; Figure 4 shows a screenshot of the results obtained in January 2017 from Google.

At first glance, the ontology appears to be available via, for example, BioPortal (20), DataHub and Bio2RDF. However, the next step would be to download MedDRA, and then we find that, while the ontology is browsable on BioPortal (the first result on our list), it is not possible to download the full ontology. Instead, BioPortal refers to the MedDRA provider site for more information on the ontology, where there is no evidence that it is available in RDF. We might still be able to download it through the BioPortal endpoint, but as well as the difficulty of exploring this option (since no explicit links or documentation are provided), we would need to assess whether this was legal, or whether it would violate the licensing terms.

Our hypothetical researcher would then move on to the other search results. The second link retrieved by Google refers to a blog post concerning MedDRA, which is not useful. We find some information about MedDRA by following the third link, but this seems to be in the context of the PharmGKB dataset and there is no evidence that the full MedDRA hierarchy is there.

In looking for the MedDRA dataset in RDF, our researcher has been forced to experiment with a variety of providers, often by querying their systems to answer questions such as the following.

- Is there a full RDF representation available, and what can actually be retrieved?
- What kind of representation is available, and how is it structured? Does it link to other data?
- Which version of MedDRA is provided?

This is a very time-consuming and error-prone process that could easily overshadow the benefits the RDF representation provides.

### YummyData’s role in fostering adoption of linked data resources

YummyData’s goal is to help foster Linked Data adoption by providing both an incentive and a medium for enhancing the quality of information offered as Linked Data. When it comes to the provision of information, our current research environment offers an incentive to provide novel data, but very little to maintain previously ‘published’ systems. Initiatives such as Elixir (https://www.elixir-europe.org/) have been started to fill this gap at the institutional level.

Our hope is that YummyData will complement such initiatives by offering providers recognition, incentive, and a way to improve the quality of their services. By measuring endpoints’ performance and their compliance with best practices, we hope to recognize the efforts of providers that have invested in maintaining their systems. By providing simple scoring (and comparison) of public endpoints, we hope to incentivize providers to invest in their systems. Finally, by providing a forum where consumers can discuss their issues, we hope to help providers improve their services. Setting up a properly maintained and documented SPARQL service is a complex process, and it is not easy for providers to evaluate where best to focus their datasets. We hope to maximize the effectiveness of datasets by enabling an evidence-based dialog between providers and consumers.

YummyData’s goals make it the natural counterpart of proposals such as FAIR, as it is able to test, assess, and monitor compliance with best practices. However, there are still limitations in how we monitor data providers that will need to be addressed in the future.

### Limitations and future developments

YummyData focuses on public life science datasets, where open monitoring (and open data ‘debugging’) is possible. One question that may need to be addressed in the future is how to extend such services to more restricted datasets, which is a general problem for Linked Data datasets. This may become increasingly relevant as data becomes more and more interlinked, even beyond the strict life science domain.

Another issue is to how to provide an ultimate assessment of data quality: YummyData can currently only provide an approximate assessment.

Although YummyData’s forum is still relatively new and underused, this depends on the visibility of the endpoints, and the feedback gathered so far has been limited to occasions when early prototype endpoints were presented. As we publicize it more widely, we expect its usage as a community resource to increase.

In addition, YummyData currently does not distinguish RDF datasets from ontologies. Since an ontology is also RDF data, this may not be a significant issue, but some providers have different policies for providing them. For example, Allie, one of the listed endpoints, supports content negotiation and provides different data formats based on abbreviation data requests, but it does not support this for ontologies since it is not useful to provide ontologies in multiple formats. In this case, YummyData may determine that Allie does not support content negotiation, so we need to consider ways of scoring or sampling URIs.

## Conclusions

We have developed a place on the Internet where data providers who make their endpoints public and their current and prospective users can communicate with each other and share knowledge. To facilitate knowledge sharing, we have proposed the Umaka Score as a means of evaluating endpoints in multiple respects, and we believe that this scoring feature will trigger increased discussion.

YummyData brings benefits to both providers and consumers. The benefits for providers include increased visibility, recognition of the quality of their implementations, and feedback on them. The benefits for users include quality-based endpoint rankings, summary information, and endpoint assessments.

We have gathered information and planted the seed of an online space for evidence-based discussion to improve the quality of RDF data provision. We hope this will be a valuable contribution to even more robust adoption of Linked Data in the life sciences, and ultimately to the improvement of data-driven life science research.

## Funding

This work was supported by the National Bioscience Database Center (NBDC) of the Japan Science and Technology Agency (JST). The original idea behind this work was discussed and implemented at the BioHackathon series of meetings.

*Conflict of interest*. None declared.

## References

[1] Bizer C., Heath T., Berners-Lee T. (2009). Linked data-the story so far. International Journal on Semantic Web and Information Systems (IJSWIS), 5, 1–22.

[2] Samwald M., Jentzsch A., Bouton C. (2011) Linked open drug data for pharmaceutical research and development. J. Cheminform., 3, 19. 21575203 10.1186/1758-2946-3-19 PMC3121711

[3] Wilkinson M.D., Dumontier M., Aalbersberg I.J. (2016) The FAIR Guiding Principles for scientific data management and stewardship. Sci. Data, 3, 10.1038/sdata.2016.18 10.1038/sdata.2016.18 PMC4792175 26978244

[4] Jupp S., Malone J., Bolleman J. (2014) The EBI RDF platform: linked open data for the life sciences. Bioinformatics, 30, 1338–1339. 24413672 10.1093/bioinformatics/btt765 PMC3998127

[5] Bushman B., Anderson D., Fu G. (2015) Transforming the medical subject headings into linked data: creating the authorized version of MeSH in RDF. J. Libr. Metadata, 15, 157–176. 26877832 10.1080/19386389.2015.1099967 PMC4749162

[6] Fu G., Batchelor C., Dumontier M. (2015) PubChemRDF: towards the semantic annotation of PubChem compound and substance databases. J. Cheminform., 7, doi: 10.1186/s13321-015-0084-4. 10.1186/s13321-015-0084-4 PMC4500850 26175801

[7] The Gene Ontology Consortium (2008) The Gene Ontology project in 2008. Nucleic Acids Res., 36, D440–D444. 17984083 10.1093/nar/gkm883 PMC2238979

[8] Croft D., O’kelly G., Wu G. (2011) Reactome: a database of reactions, pathways and biological processes. Nucleic Acids Res., 39, D691–D697. 21067998 10.1093/nar/gkq1018 PMC3013646

[9] Caspi R., Billington R., Ferrer L. (2016) The MetaCyc database of metabolic pathways and enzymes and the BioCyc collection of pathway/genome databases. Nucleic Acids Res., 44, D471–D480. 26527732 10.1093/nar/gkv1164 PMC4702838

[10] The UniProt Consortium (2017) UniProt: the universal protein knowledgebase. Nucleic Acids Res., 45, D158–D169. 27899622 10.1093/nar/gkw1099 PMC5210571

[11] Cerami E.G., Gross B.E., Demir E. (2010) Pathway Commons, a web resource for biological pathway data. Nucleic Acids Res., 39, D685–D690. 21071392 10.1093/nar/gkq1039 PMC3013659

[12] Digles D., Zdrazil B., Neefs J.M. (2016) Open PHACTS computational protocols for in silico target validation of cellular phenotypic screens: knowing the knowns. Med. Chem. Commun., 7, 1237–1244. 10.1039/c6md00065g PMC5063042 27774140

[13] Galperin M.Y., Fernández-Suárez X.M., Rigden D.J. (2017) The 24th annual Nucleic Acids Research database issue: a look back and upcoming changes. Nucleic Acids Res., 45, D1–D11. 28053160 10.1093/nar/gkw1188 PMC5210597

[14] Bhardwaj A., Bhattacherjee S., Chavan A. (2014) Datahub: Collaborative data science & dataset version management at scale. arXiv preprint arXiv:1409.0798.

[15] Tummarello G., Delbru R., Oren E. (2007) Sindice.com: weaving the Open Linked Data. *The Semantic Web*. Lecture Notes in Computer Science, 4825, 552–565.

[16] Bolleman J.T., Mungall C.J., Strozzi F. (2016) FALDO: a semantic standard for describing the location of nucleotide and protein feature annotation. J. Biomed. Seman., 7, 10.1186/s13326-016-0067-z 10.1186/s13326-016-0067-z PMC4907002 27296299

[17] Katayama T., Wilkinson M.D., Aoki-Kinoshita K.F. (2014) BioHackathon series in 2011 and 2012: penetration of ontology and linked data in life science domains. J. Biomed. Seman., 5, 5. 10.1186/2041-1480-5-5 PMC3978116 24495517

[18] Yamaguchi A., Kozaki K., Lenz K. (2016) Semantic Data Acquisition by Traversing Class-Class Relationships Over Linked Open Data. In: Li Y.F. (eds). *Semantic Technology. JIST 2016*. Lecture Notes in Computer Science, Springer, Cham, Vol.10055, pp. 136–151.

[19] Ruttenberg A., Rees J.A., Samwald M., Marshall M.S. (2009) Life sciences on the Semantic Web: the Neurocommons and beyond. Brief. Bioinform., 10, 193–204. 19282504 10.1093/bib/bbp004

[20] Whetzel P.L., Noy N.F., Shah N.H. (2011) BioPortal: enhanced functionality via new Web services from the National Center for Biomedical Ontology to access and use ontologies in software applications. Nucleic Acids Res., 39, W541–W545. 21672956 10.1093/nar/gkr469 PMC3125807

